# Quantum Effects In Imaging Nano-Structures Using Photon-Induced Near-Field Electron Microscopy

**DOI:** 10.1038/s41598-019-42624-w

**Published:** 2019-04-16

**Authors:** Naglaa Etman, Afaf M. A. Said, Khaled S. R. Atia, Reem Sultan, Mohamed Farhat O. Hameed, Muhamed Amin, S. S. A. Obayya

**Affiliations:** 1 Center for Photonics and Smart Materials, Zewail City of Science and Technology, October Gardens, Giza, 12578 Egypt; 2 Electronics and Communications Engineering Department, Faculty of Engineering, Mansoura University, Mansoura, 35516 Egypt; 3 Advanced Research Complex, University of Ottawa, Ottawa, ON K1N 6N5 Canada; 4 Nanotechnolgy Engineering Program, University of Science and Technology, Zewail City of Science and Technology, October Gardens, Giza, 12578 Egypt; 5 Mathematics and Engineering Physics Dept., Faculty of Engineering, Mansoura University, Mansoura, 35516 Egypt; 6 Center for Free-Electron Laser Science, Deutsches Elektronen-Synchrotron DESY, Notkestrasse 85, 22607 Hamburg, Germany; 7 Department of Sciences, University College Groningen, Hoendiepskade 23/24, 9718 BG Groningen, Netherlands

## Abstract

In this paper, we introduce the quantum mechanical approach as a more physically-realistic model to accurately quantify the electron-photon interaction in Photon-induced near-field electron microscopy (PINEM). Further, we compare the maximum coupling speed between the electrons and the photons in the quantum and classical regime. For a nanosphere of radius 2.13 *nm*, full quantum calculations show that the maximum coupling between photon and electron occurs at a slower speed than classical calculations report. In addition, a significant reduction in PINEM field intensity is observed for the full quantum model. Furthermore, we discuss the size limitation for particles imaged using the PIMEN technique and the role of the background material in improving the PINEM intensity. We further report a significant reduction in PINEM intensity in nearly touching plasmonic particles (0.3 *nm* gap) due to tunneling effect.

## Introduction

Different electron microscopy techniques^1–5^ gain a lot of interest nowadays due to their ability to image at higher resolution than light microscopes limited by diffraction effect. Photon-induced near-field electron microscopy (PINEM)^6–8^ is one of the most recent techniques using the coupling between the electron and the optical near field around the nanostructures. Dissimilar to the transmission electron microscopy^9^ using swift electron for imaging, PINEM uses ultra-short laser pulse to excite the near field, then fast electrons are sent for probing. The mechanical work performed by the scattered field on the swift electrons is then measured^6–8,10^. Recently, PINEM showed a great potential for imaging high resolution nanostructures and visualizing phenomena such as plasmmonics in both time and space^11^.

The heart of PINEM technique is the electron-photon interactions, which are forbidden in free space due to the mismatch in momentum^3,7^. However, the spatial confinement Δ*z* caused by the scattered near field of the imaged nanostructure alters the electrons momentum^11^. Hence, the coupling between the photon and electrons takes place. Although there is an upper limit for the particle size that can be imaged with PINEM, this limit could be tuned by using different dielectric-background materials. Theoretically, there is thus no lower limit for the particle size^3^. However, small particle size produces lower scattered field intensity with smaller spatial confinement and hence smaller PINEM field. In addition, the maximum coupling spatial frequency would be shifted. This is because PINEM field is actually the Fourier transform of the scattered electric field component in the direction of propagation at the spatial frequency *ω*/*v*^7,10^,

1 $${F}_{z}(x,y)={\int }_{-\infty }^{+\infty }{E}_{z}(x,y,z){e}^{-i(\frac{\omega }{v})z}dz.$$

Therefore, a spread in the spatial frequency domain and a shift towards the higher frequencies are expected for small spatial confinement. The decrease in the amplitude of PINEM field is a consequence to the spread in frequency domain. Thus, understanding the correlation between the photon, electron and the size of the nanostructure is essential to understand the capabilities of PINEM technique.

PINEM may provide valuable contribution to our understanding of plasmonics which nowadays receives high interest due to its capability of subwavelength localization beyond the diffraction limit^12^. Generally, the essential properties of plasmonic nanostructures can be theoretically characterized just by solving the classical Maxwell’s equations^13^. However, for nanostructures with a few nanometers radii of curvature and/or with subnanometer separation between particles, the classical treatment completely fails, even if the non-local nature is included^14–17^. Because non-local optics neglects electron-density spill out^14^, the inhomogeneity of the electron density at the surface of a metal surrounding by a dielectric material. Thus, classical calculations cannot predict the correct Surface Plasmon Resonance (SPR) for isolated small nanoparticles^14,18^. Moreover, recent optical experiments on two plasmonic nanoparticles separated by a few subnanometer completely deviate from classical predictions^19^. The overlap of electron densities and the tunnelling current in such subnanometer gaps extremely affect the optical response. Thus, classical methods are inadequate, and considering such quantum effects using methods like Time-Dependent Density Functional Theory (TDDFT)^20,21^ gains a lot of interest in the field of nanoplasmonics^22^. Despite of the high precision of such full quantum solvers, they require huge computational resources in terms of time and memory. Therefore, many research efforts have been recently paid to introduce new Quantum Corrected Models (QCM)^15,19,23^ which could modify any classical electromagnetic framework to take into account such photoinduced tunnel current between the two plasmonic nanoparticles separated by a few subnanometer gaps but with incomparable computational efforts. So, to accurately calculate scattered electric field which is a vital parameter in PINEM calculations, quantum analysis should be considered. Moreover, our theoretical study here shows promise that PINEM would be used for visualizing such quantum effects in reality.

In this paper, we introduce, for the first time to the best of our knowledge, full quantum analysis to calculate PINEM field integral *F*_*z*_ for a nanoparticle of radius = 2.13 *nm*. Then, we compare the quantum results obtained by solving the Khon-Sham (KS) equation with those obtained by using classical electrodynamics. Next, the correlation between the size of a various spherical nanostructures and the electron-photon coupling speed is studied in different dielectric mediums. In addition, the QCM^15,23,24^ is used to investigate the effect of electron tunneling on the electron-photon coupling and hence on PINEM intensity. Further, we demonstrate that PINEM technique can prove the tunneling process at very small gap sizes (few Ångsrtöm separations).

## Results

It is well known that the momentum of the electron is changed by the number of photons absorbed (energy gain) or emitted (energy loss) via^3^ Δ*p* = (2*mE* ± 2*mħωn*)^1/2^ − (2*mE*)^1/2^, where m is the electron mass, E is its initial energy, *ħ* is the reduced Planck constant, *ω* is the photon angular momentum, and n is the number of photons. Due to the large difference between photon and electron energies, the change in momentum can be approximated as Δ*p* ≈ ±*nħω*/*v* where *v* is the electron’s speed. The Heisenberg uncertainty principle relates the change in momentum with the spatial confinement caused by nanoparticle via (Δ*p*Δ*z* ~ *h*). For Δ*p* ≈ ±*nħω*/*v* to hold, and for only one photon, n = 1, a spatial confinement of Δ*z* = 2*π*/*ω* is required for the coupling process. For instance, a photon with energy 2.4 *eV* and electrons with *v*_*e*_ = 0.7*c* (200 *KeV*), where *c* is the speed of light in free space, Δ*z* equals 350 *nm*. To couple more photons (*n* > 1), the spatial confinement is less than 350 nm.

### Na-cluster

A single nanoparticle of Sodium (Na) with radius ~2 *nm* is thus considered here. The electron-density spill out^14^ of such particle size (a few nanometer radii ~2 *nm*) dramatically changes the absorption properties and hence on the scattered field. That is why, it is essential to introduce here a full quantum simulation of PINEM intensity while studying such plasmonic nanoparticle. The Quantum calculations are performed with the Octopus package^25^. Octopus solves the KS time dependent equations in a real-space representation based on TDDFT which quantum-mechanically describes electrons. Typical convergence parameters are spatial-grid spacing of 0.26 *Å*, total propagation time *T*_*max*_ = 40*ħ*/*eV*, and time step *dt* = 0.0025*ħ*/*eV*. Dissimilar to noble metals, the core electrons in Sodium-cluster do not need to be clearly considered to evaluate the optical properties^14^. Moreover, the atom- and jellium-TDDFT results are fairly similar but with incomparable computational cost^14^. In addition, jellium model has good agreements with experiments^14,26,27^, besides its simplicity^28^. Therefore, we use the spherical jellium model^14^ for describing the spherical cluster of *Na*_*N*_ with *N* = 1074 atoms. The Wigner-Seitz radius of Na is *r*_*s*_ = 3.93 Bohr raduis^14^, so the jellium sphere radii is *R* ≈ 2.13 *nm*.

First, the ground state (gs) of the spherical *Na*_1074_ jellium cluster is calculated. Next, we use Delta kick excitation to obtain the linear optical absorption spectrum from which the resonance frequency is extracted at *ω*_0_ = 3.3 *eV*. Then, the time-propagation with 16384 iterations is used to excite the Na nanoparticle by an x-polarized photon propagating in z-direction at *ω*_0_ = 3.3 *eV* with *E*_0_ amplitude. We extract the Hartree potential (*v*_*Hartree*_) for the *xz*–plane at *y* = 0. Then, the scattered electric field |*E*_*z*_|/*E*_0_ results from the gradient of *v*_*Hartree*_ extracted. For the PINEM calculations, an electron is incident in z-direction to interact with the scattered field of the nanoparticle. More details about the quantum calculations are included in the Supplementary File.

We perform the quantum simulation on the Bibliotheca Alexandrina High-Performance Computing cluster (BA-HPC) which is with 100 TFLOPS, 98 servers, 2 CPU per server, 128 GB RAM/server, 1,968 CPU-cores, 40 Gbps interconnect bandwidth and 2 General-Purpose Graphics Processing Units accelerators (GPGPUs) per server. Although jellium model is preserved, and the simulation is distributed on 240 slots of the BA-HPC, the computational cost is still huge. It takes more than two months, 61-3:23:33 (the jobs elapsed time), only for running the time-propagation step without the gs or the Delta kick excitation. We need to write the huge simulation results after each 8 iterations to have a good resolution time domain data for the Fourier transforms; however, that required huge memory and space for processing and saving [16384 iterations/8 iterations × 32265 × 3] *v*_*Hartree*_ matrix size. We choose this huge particle size, 2 *nm*, from quantum point of view to be visible for the classical simulator. That is why we settle for studying only Na.

**Figure 1:**
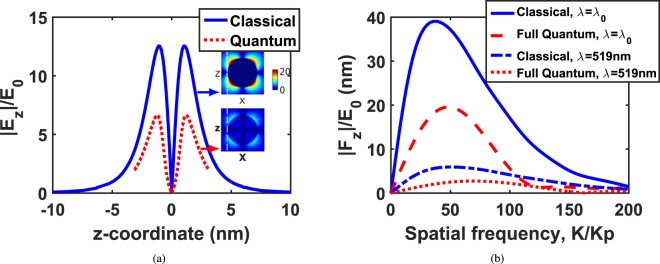
Quantum and classical calculations for: (**a**) |*E*_*z*_|/*E*_0_ at the tangential cutline of the Na sphere and (**b**) its |*F*_*z*_|/*E*_0_ while changing *K*/*K*_*p*_, where *K*_*p*_ is the spatial frequency of photon. The insets display |Ez|/E0 in xz-plane at y = 0 for the Na-cluster.

We notice that the quantum resonance frequency is shifted with respect to the classical simulation, where its *ω*_0_ = 3.24 *eV*. Because classical theory considers the boundaries between metal and a surrounding dielectric medium as sharp-boundaries neglecting the vital electron-density spill out of such nanoparticle^14^. Therefore, a reduction in the quantum scattered electric field (|*E*_*z*_|/*E*_0_) could be observed in Fig. 1(a) with respect to the classical one. Consequently, the quantum PINEM field (|*F*_*z*_|/*E*_0_) is also smaller than that obtained by classical calculations at *ω*_0_ illumination as shown in Fig. 1(b). Moreover, Fig. 1(b) shows that the resonance spatial frequency (*K*/*K*_*p*_), at which PINEM field is maximum, is shifted to the higher direction for quantum results than those obtained by classical calculations. On the other hand, when we use a 519 *nm* illumination, as widely used in PINEM technique^6–8,10^, the scattered field and hence the PINEM field shown in Fig. 1(b) dramatically decreases, as expected. Because 519 *nm* is extremely shifted with respect to the resonance frequency of the Na cluster we study. Further, Fig. 1(b) shows that the quantum resonance spatial frequency is still shifted comparable with the classical calculations. A possible way to increase PINEM field is to use a higher index dielectric material as a surrounding medium which decreases the photon velocity and achieves the conservation of angular momentum between photon and electron as discussed later.

### PINEM maximum coupling

**Figure 2:**
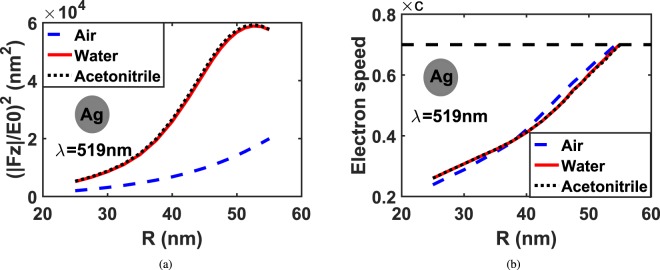
For different background materials: (**a**) Maximum PINEM intensity computed for different particle size of silver sphere *n* = 0.05 + 3.31*i* at 519 *nm* illumination. (**b**) Electron speeds at maximum PINEM field.

To seek PINEM maximum coupling and its limitations with respect to the particle size and the background material, we study silver particles with different sizes, from 25 *nm* to 55 *nm* radius, excited by 519 *nm* wavelength photon in different background material. Figure 2(a) shows the relation between the particle size and the maximum achievable PINEM intensity (|*F*_*z*_|^2^)^29,30^ normalized to the input power.

It may be observed that PINEM intensity increases with increasing the particle size in all different background materials. This is because PINEM intensity is directly proportional to the electric field component in z-direction. Further, the scattering cross-section *σ*_*scat*_ is also directly proportional to the particle size (*σ*_*scat*_ ∝ *R*^6^) according to Rayleigh theory^31,32^. Hence, PINEM intensity increases with the increase of the particle size. Also, PINEM intensity is much higher in water, *n* = 1.33, and Acetonitrile, *n* = 1.34, than air, *n* = 1. According to Rayleigh scattering, the scattering cross-section is directly proportional to the difference between the permittivity of the background material and the permittivity of the particle size (*σ*_*scat*_ ∝ |Δ*ε*|^2^). Therefore, different background materials than air can produce stronger scattered field |*E*_*z*_|/*E*_0_ and hence higher PINEM intensity. Figure 2(b) shows the maximum coupling speed on the electron for different particle sizes in different background materials (air, water, and Acetonitrile). Since, the electron speed cannot reach the speed of light in free space, we limited our calculation to a maximum electron speed of *v*_*e*_ = 0.7*c*. Therefore, it may be seen that the upper limit of particle radius that can be imaged using PINEM under these conditions is 54 *nm* in air and ~55 *nm* in water and Acetonitrile. This limit differs slightly in water and Acetonitrile because of the strong field confinement through background materials with high refractive indices than air.

**Figure 3:**
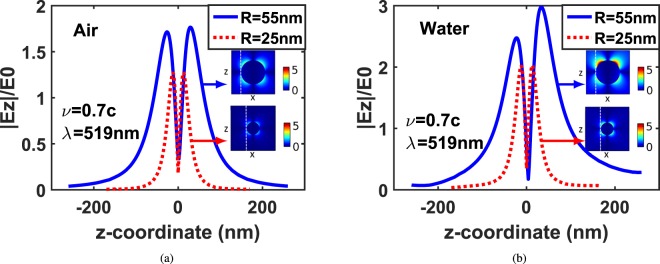
|*E*_*z*_|/*E*_0_ at the edge of the silver sphere, for different particle sizes at back-ground materials: (**a**) Air and (**b**) Water. The insets display (|*E*_*z*_|/*E*_0_) in xz-plane for each particle.

Figure 3 compares |*E*_*z*_|/*E*_0_, at the edge of the nanostructure for silver sphere of 55 *nm* and 25 *nm* radius placed in air and water. In the case of air dielectric, the scattered electric field components are quite symmetric regarding the z-coordinates. This is due to the particle size which is much smaller than the excitation wavelength (*R* < *λ*/10). Hence, Rayleigh scattering occurs where the scattered field is symmetric. Whereas, in the case of the water dielectric, the electric field is asymmetric in the z-coordinates because the particle size becomes higher than *λ*_*eff*_/10 where *λ*_*eff*_ = *λ*/*n*. Therefore, the scattering follows Mie scattering instead of Rayliegh scattering where the scattered field becomes asymmetric. In contrast, at the small particle size of radius 25 *nm*, |*E*_*z*_|/*E*_0_ along the electron trajectory is symmetric in both medium air and water. Also, it can be noticed that |*E*_*z*_|/*E*_0_ is greater in water than in air.

### PINEM and Tunneling

**Figure 4:**
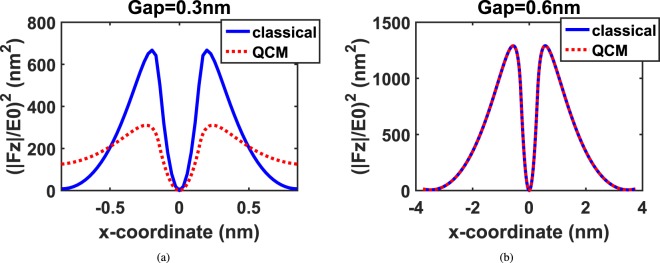
Quantum and classical (|*F*_*z*_|/*E*_0_)^2^ along x-axis for *y* = 0 plane of two 50 *nm* silver nanoparticles with gap sizes: (**a**) (0.3 *nm*) and (**b**) (0.6 *nm*) placed in air.

In the attempt to study the effect of nearly touching plasmonic particles on PINEM intensity (|*F*_*z*_|^2^), we consider two silver spheres of radius 50 *nm* placed in x-direction separated by a gap. For a gap size of 0.3 *nm*, where the tunneling is more likely to happen, Fig. 4(a) shows PINEM intensity calculated of the xz-plane at *y* = 0 for the two nanoparticles using classical theory and QCM.

**Figure 5:**
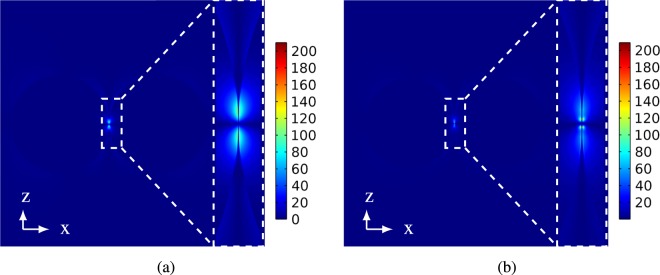
|*E*_*z*_|/*E*_0_ in xz-plane at *y* = 0 for the two 50 *nm* silver nanoparticles with gap sizen 0.3 *nm* calculated by (**a**) classical theory and (**b**) QCM, respectively. The insets Focus on a cross section of |*E*_*z*_|/*E*_0_ at the gap between the two particles.

It may be observed from Fig. 4(a) PINEM intensity calculated using QCM is less than PINEM intensity calculated using classical theory. In the classical theory, the tunneling cannot be described. Therefore, it may be noted from Fig. 5(a) that there is a field enhancement in the gap between the two nanoparticles.

**Figure 6:**
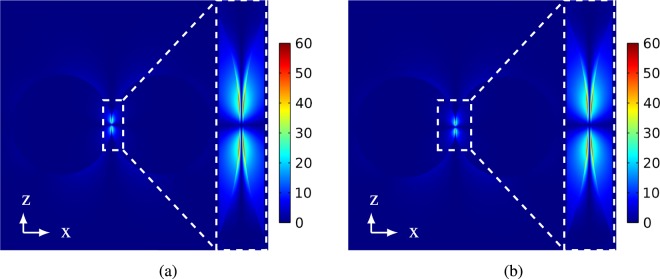
|*E*_*z*_|/*E*_0_ in xz-plane at *y* = 0 for the two 50 *nm* silver nanoparticles with gap size 0.6 *nm* calculated by: (**a**) Classical theory and (**b**) QCM, respectively. The insets Focus on a cross section of |*E*_*z*_|/*E*_0_ at the gap between the two particles.

However, this is not true because the charge transfer between the two nanoparticles makes the gap conductive. Thus, there should be degradation in the field intensity in the gap due to losses, and hence PINEM intensity should be decreased. On contrast, the QCM treats the gap between the two nanoparticles as a conductive gap and hence takes the tunneling phenomena into consideration^15,23^. This tunneling effect considered by QCM appears clearly in the reduction in the scattered field shown in Fig. 5(b). On the other side, where the tunneling becomes less likely to happen for 0.6 *nm* gap, Fig. 4(b) shows a good agreement between the classical results and the QCM results. This is because the gap becomes less conductive, and hence the field enhancement computed using the classical theory and QCM is the same as shown in Fig. 6(a,b), respectively. Therefore, we expect that PINEM technique can be used to visualize the tunneling process at very small gap sizes. For the effect of tunneling over the entire space, the same study is repeated for two 50 nm sodium particles in the Supplementary File. More information is also included in the Supplementary File for how PINEM calculation is extended in real space and spatial frequency.

## Supplementary information

SUPPLEMENTARY INFO Quantum Effects In Imaging Nano-Structures Using Photon-Induced Near-Field Electron Microscopy
