# Supplementary material for: Quantum Effects In Imaging Nano-Structures Using Photon-Induced Near-Field Electron Microscopy
Source: Sci Rep. 2019 Apr 16;9:6139. doi: 10.1038/s41598-019-42624-w (PMC6468085; doi:10.1038/s41598-019-42624-w)
Supplement: Supplementary file 1 — SUPPLEMENTARY INFO Quantum Effects In Imaging Nano-Structures Using Photon-Induced Near-Field Electron Microscopy [file 41598_2019_42624_MOESM1_ESM.docx]

**Supplementary materials: Quantum Effects In Imaging Nano-Structures Using Photon-Induced Near-Field Electron Microscopy**

**Naglaa Etman**^1,2,+^**, Afaf M.A. Said**^1,+^**, Khaled S.R. Atia**^1,3,+^**, Reem Sultan**^2,+^**, M.F.O.Hameed**^1,4,5,*^**, Muhamed Amin**^6,**^**, and S.S.A.Obayya**^1,***^

^1^Center for Photonics and Smart Materials, Zewail City of Science and Technology, Giza, 12578, Egypt

^2^Electronics and Communications Engineering Department, Faculty of Engineering, Mansoura University,

Mansoura 35516, Egypt

^3^Advanced Research Complex, University of Ottawa, ON K1N 6N5, Canada

^4^Faculty of Engineering, Mansoura University, Mansoura 35516, Egypt

^5^Nanotechnolgy Engineering Program, University of Science and Technology, Zewail City of Science and Technology, October Gardens, 6th of October City, Giza, Egypt

^6^Center for Free-Electron Laser Science, Deutsches Elektronen-Synchrotron DESY, Notkestrasse 85, 22607 Hamburg, Germany

^*^corresponding.mfarahat@zewailcity.edu.eg

^**^corresponding.muhamed.amin@cfel.de

^***^corresponding.sobayya@zewailcity.edu.eg

^+^these authors contributed equally to this work

**0.1 Quantum calculations details for Na-cluster:**

For our quantum calculations of the Sodium-cluster of 2.13nm, the time-propagation with 16384 iterations is used to excite the Na nanoparticle by an x-polarized photon propagating in z-direction at $\omega$_0_ = 3.3eV with E_0_ amplitude. Fig.S1 shows the input laser pulse used for the excitation.

| **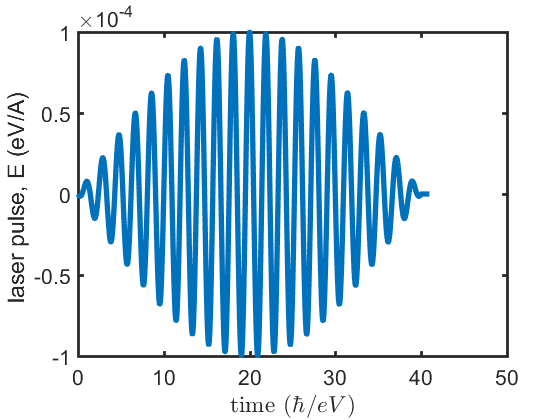**  **Fig. S1: The input laser pulse used to excite the Na-cluster, x-polarized source propagating in z-direction at** $\boldsymbol{\omega}$**_0_ = 3.3eV with E0 amplitude.** |
| --- |

Then, we extract the Hartree potential (V_Hartree_) for the xz-plane at y = 0. After Fourier transform, Fig.S2 and Fig.S3 show V_Hartree_ (eV) in frequency domain**.**

| 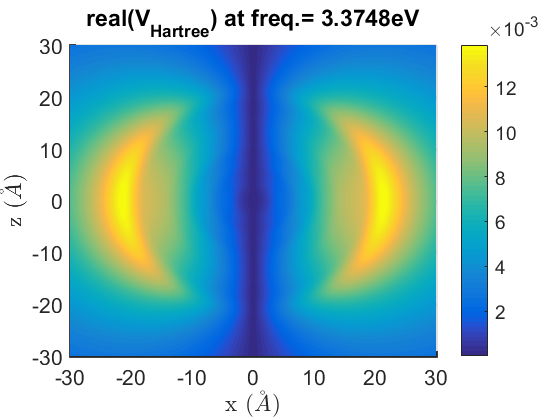  **Fig. S2: the real part of Hartree potential (V_Hartree_) for the xz-plane at y = 0 of the Na-cluster at frequency =3.3746 eV.** |
| --- |

| 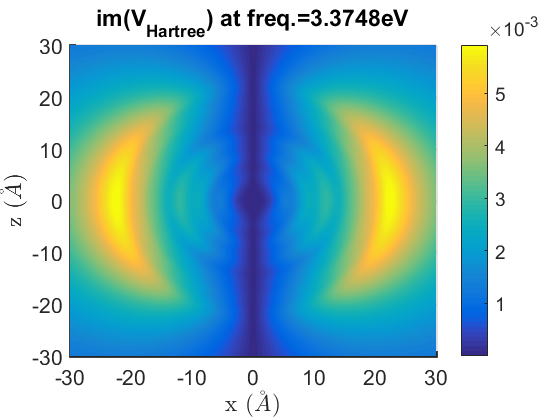  **Fig. S3: The imaginary part of Hartree potential (V_Hartree_) for the xz-plane at y = 0 of the Na-cluster at frequency =3.3746 eV.** |
| --- |

Then, the scattered electric field |Ez|\E0 results from the gradient of V_Hartree_ extracted. Fig. S4 show the absolute induced electric fields, transverse |Ex| and scattered |Ez| components, respectively, at frequency =3.3746 eV.

| **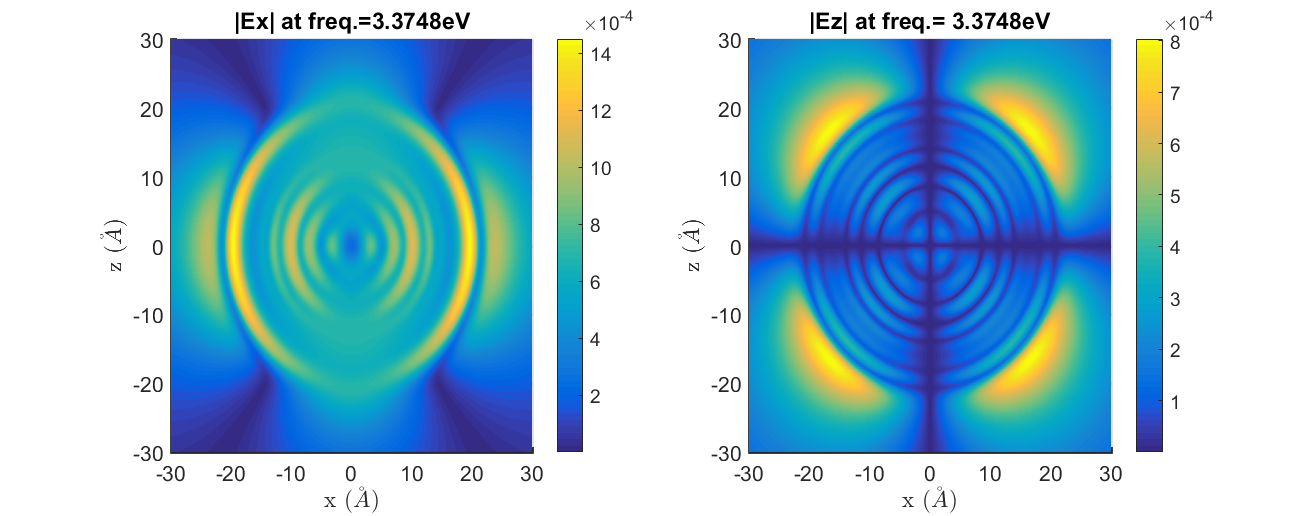**  **Fig. S4: The absolute induced electric fields, transverse \|Ex\| and scattered \|Ez\| components, respectively, for the Na-cluster at frequency =3.3746 eV.** |
| --- |

**0.2 Extending PINEM calculations in real space and spatial frequency domain:**

For the PINEM calculations, an electron is incident in z-direction to interact with the scattered field of the nanoparticle. Fig. S5 shows 2D image for the PINEM field of the Na-cluster as a function of the space, x-direction, and the spatial frequency, K/Kp. This figure indicates that PINEM can correctly determine the cluster size around K/Kp ~40.

| 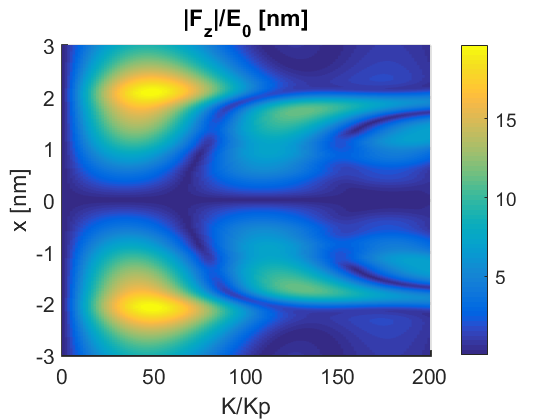  **Fig. S5: 2D image for the PINEM field of the Na-cluster illuminated by** $\boldsymbol{\omega}$**_0_ = 3.3eV as a function of the space, x-direction, and the spatial frequency, K/Kp.** |
| --- |

In order to extend the PINEM calculations and not to seem limited in space, Fig. S6 shows 2D image for the PINEM field of the Ag-dimers with gap=0.6nm as a function of the space, x-direction, and the spatial frequency, K/Kp. This figure indicates that PINEM can clearly image the too small gap between the two particles at a little bit high K/Kp (around ~15).

| 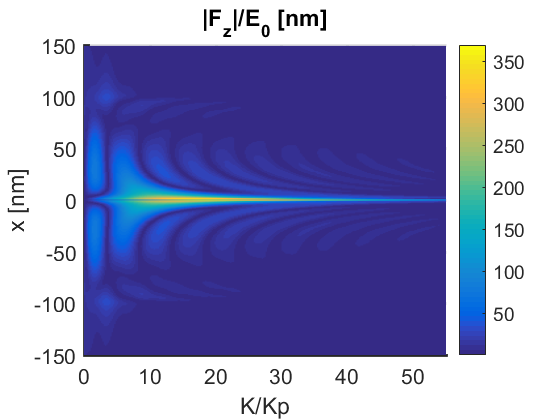  **Fig. S6: 2D image for the PINEM field of the Ag-dimers with gap=0.6nm as a function of the space, x-direction, and the spatial frequency, K/Kp.** |
| --- |

**0.3 Quantum-corrected model Details**

| **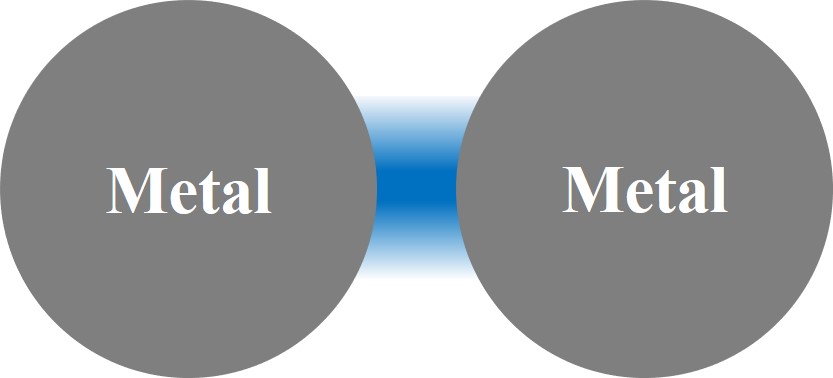** |
| --- |
| **Fig. S7: Schematic diagram of two nearly touching metal particles** |

Figure S7 shows a schematic of the quantum-corrected model where the two particles and the surrounding vacuum are modeled with abrupt interfaces everywhere except the gap between the particles. The permittivity of the metal particles is described by Drude model as

$$\varepsilon_{m}=\varepsilon_{\infty}-\frac{\omega_{p}^{2}}{\omega\left( \omega+i\gamma_{p} \right)}$$

where $\omega_{p}$ is the plasma frequency, $\gamma_{p}$ is the damping frequency, and $\varepsilon_{\infty}$ is dielectric response when the frequency $\omega$ approaches infinity. The shaded area in the gap between the two particles as shown in Fig. S7 represents a fictious material introduced to account the charge transfer between the particles due to quantum mechanical tunneling. This material is defined by Drude model as

$$\varepsilon_{g}=\varepsilon_{g\infty}-\frac{\omega_{g}^{2}}{\omega\left( \omega+i\gamma_{g} \right)}$$

Where $\varepsilon_{g\infty}$ and $\omega_{g}$ are assumed to equal $\varepsilon_{\infty}$ and $\omega_{p}$, respectively. Whilst, the damping rate $\gamma_{g}$ is a function of the lateral distance between the particles which can be obtained from the static conductivity $\sigma_{0}$ as

$$\gamma_{g}=\frac{\omega_{g}^{2}}{4\pi\sigma_{0}}$$

The static conductivity is then calculated by integrating the electron tunneling (T) in the gap up to the Fermi level $\Omega_{f}$ as

$$\sigma_{0}=l\frac{2}{\left( 2\pi\right)^{2}}\int_{\Omega_{0}}^{\Omega_{f}} T\left( \Omega\right)d\Omega$$

where $l$ is the gap size. The electron tunneling is computed by solving the basic Schrödinger equation [S1].

It is previously reported that the calculation of the damping frequency $\gamma_{g}$ by solving Schrödinger equation matches the full quantum calculations for bigger gap sizes ($l>3A^{o}$). However, the continuity of the damping frequency values at different gap sizes makes evaluating $\gamma_{g}$ at smaller gaps plausible via interpolation since $\gamma_{g}$ for $l=0$ (no gap) approaches $\gamma_{p}$ and the values of $\gamma_{g}$ for large gap sizes matches the full quantum calculations.

The potential barrier between the two metal particles can be expressed as reported in [S1]. Figure S8(a) and (b) show the potential barrier formed between two silver and sodium particles, respectively.

| 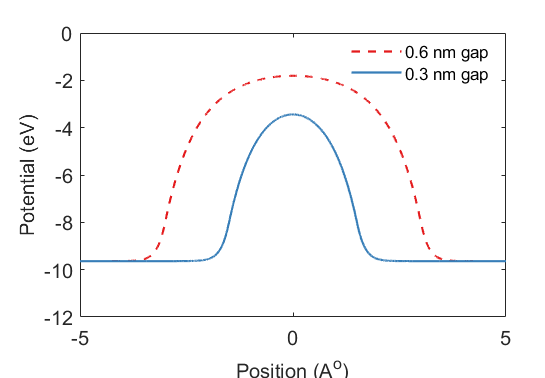 | 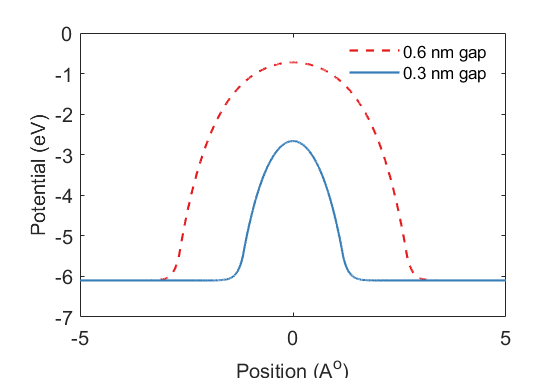 |
| --- | --- |
| (a) Ag | (b) Na |
| **Fig. S8: 2D the potential across 0.3 nm and 0.6 nm gaps for (a) silver and (b) sodium.** | |

The Drude model parameters for silver and sodium are present in Table. S1.

**Table S1. the Drude model parameters for silver, and sodium.**

|  | $\omega_{p}[eV]$ | $\gamma_{p}$ |
| --- | --- | --- |
| Silver (Ag) | 9.065 | 0.0212 |
| Sodium (Na) | 5.16 | 0.218 |

The values of the refractive indices (n) of the fictious material for silver particle are present in Table S2 for different gap sizes. Table S3 contains the refractive indices for different gapes between two sodium particles. The values are computed using two approaches. The first approach is the termed the long-range approach where the simple Schrödinger equation is solved for the potential barrier formed between the two metal barriers. The second approach is named exponential fit where the damping rate is computed for bigger gap sizes ($l>0.3 nm$) and then fitted to exponential relation so that is approached the metal damping rate at $l=0$.

**Table S2 the refractive indices of the fictious material for different gaps between two silver particles at 519 nm.**

| Gap size (Å) | $n$ (long range) | $n$ (exponential fit) |
| --- | --- | --- |
| 1 | -- | 0.1898 - 3.6949i |
| 2 | -- | 1.3166 - 2.7989i |
| 3 | -- | 1.1088 - 0.5878i |
| 4 | 1.0001 - 0.0120i | 1.0013 - 0.0607i |
| 5 | 1.0000 - 0.0018i | 1.0000 - 0.0056i |
| 6 | 1.0000 - 0.0002i | 1.0000 - 0.0005i |
| 7 | 1.0000 - 0.0000i | 1.0000 - 0.0000i |
| 8 | 1.0000 - 0.0000i | 1.0000 - 0.0000i |
| 9 | 1.0000 - 0.0000i | 1.0000 - 0.0000i |
| 10 | 1.0000 - 0.0000i | 1.0000 - 0.0000i |

**Table S3 the refractive indices of the fictious material for different gaps between two sodium particles at 519 nm.**

| Gap size ($Å$) | $n$ (long range) | $n$ (exponential fit) |
| --- | --- | --- |
| 1 | -- | 0.3956 - 1.8228i |
| 2 | -- | 0.9074 - 1.2392i |
| 3 | -- | 1.0091 - 0.4460i |
| 4 | 1.0000 - 0.0002i | 1.0010 - 0.1229i |
| 5 | 1.0000 - 0.0000i | 1.0001 - 0.0325i |
| 6 | 1.0000 - 0.0000i | 1.0000 - 0.0086i |
| 7 | 1.0000 - 0.0000i | 1.0000 - 0.0023i |
| 8 | 1.0000 - 0.0000i | 1.0000 - 0.0006i |
| 9 | 1.0000 - 0.0000i | 1.0000 - 0.0002i |
| 10 | 1.0000 - 0.0000i | 1.0000 – 0.0000i |

It may be noted that for the two approaches the long range and the exponential fit, the imaginary part which corresponds to the charge transfer between the two particles approaches zero for bigger gaps as the tunneling effect is reduced. Also, since the background material used in this simulation is air, the real part approaches unity for bigger gaps.

**0.4 PINEM and Tunneling**

we consider two sodium spheres of radius 50nm placed in x-direction separated by a gap. For a gap size of 0.3 nm, where the tunneling is more likely to happen, Fig.9(a) shows PINEM intensity calculated of the xz-plane at y = 0 for the two nanoparticles using classical theory and QCM. It may be observed from Fig.S9(a) PINEM intensity calculated using QCM is less than PINEM intensity calculated using classical theory. In the classical theory, the tunneling cannot be described. Therefore, it may be noted from Fig.S10(a) that there is a field enhancement in the gap between the two nanoparticles.

| 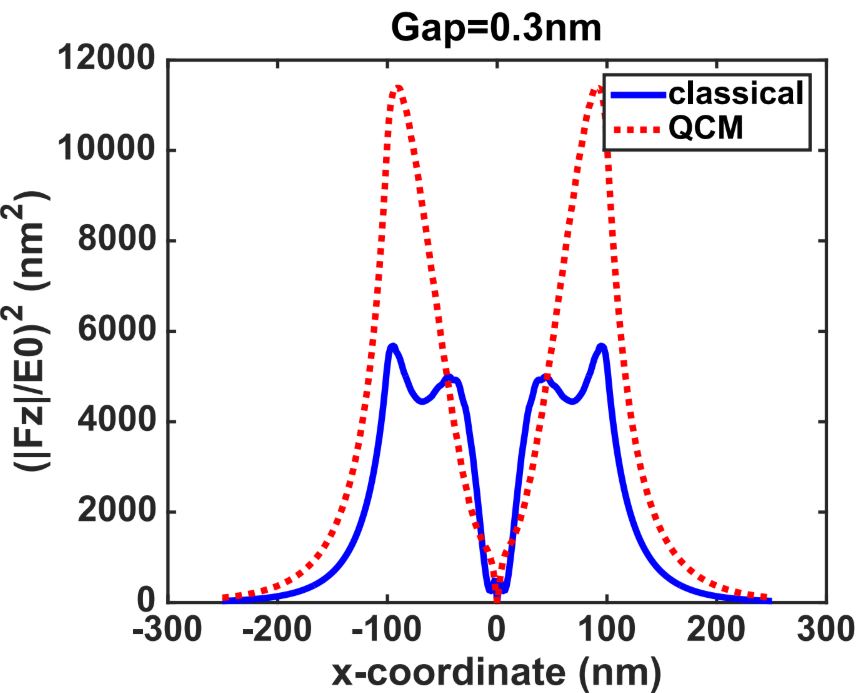 | 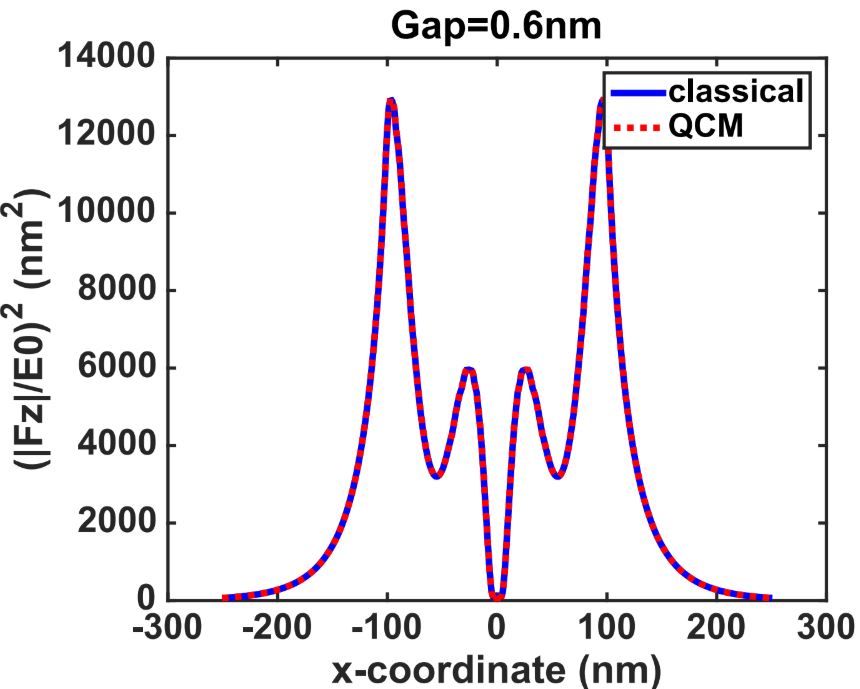 |
| --- | --- |
| (a) | (b) |
| **Fig. S9: Quantum and classical** ${\boldsymbol{(\vert}\boldsymbol{F}_{\boldsymbol{z}}\boldsymbol{\vert/E}\boldsymbol{0)}}^{\boldsymbol{2}}$**along x-axis for y = 0 plane of two 50nm sodium nanoparticles with gap sizes: (a) (0:3nm) and (b) (0:6nm) placed in air.** | |

| 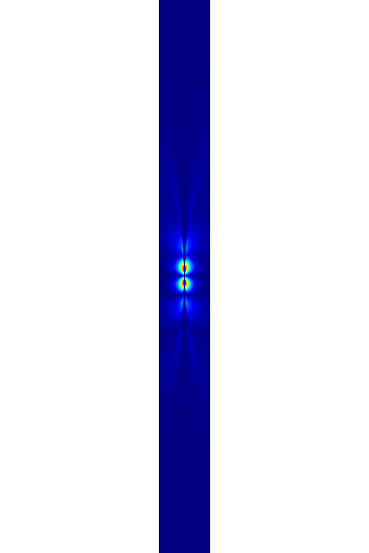 | 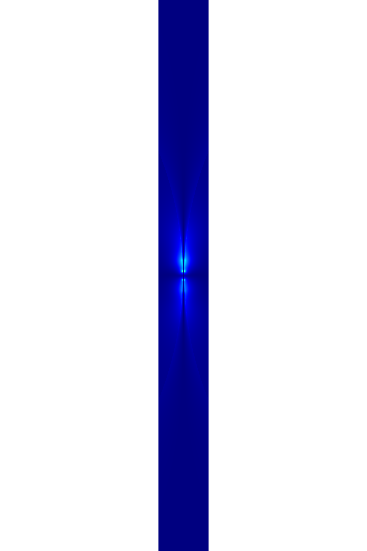 |
| --- | --- |
| (a) Classical | (b) QCM |
| **Fig. S10:** $\boldsymbol{\vert}\boldsymbol{F}_{\boldsymbol{z}}\boldsymbol{\vert/E}\boldsymbol{0}$ **in xz-plane at y = 0 for the two 50nm silver nanoparticles with gap sizen 0:3nm calculated by (a) classicaltheory and (b) QCM, respectively. The inset Focus on a cross section of jEzj=E0 at the gap between the two particles.** | |

On the other side, The tunneling effect considered by QCM appears clearly in the reduction in the scattered field shown in Fig.S10(b) . For the 0.6 nm gap, the effect of the tunneling diminishes and therefore the PINEM intensities computed classically and using the QCM are in perfect agreement as shown in Fig.S9(b).

References

[S1] Esteban, R., Zugarramurdi, A., Zhang, P., Nordlander, P., García-Vidal, F. J., Borisov, A. G., & Aizpurua, J. (2015). A classical treatment of optical tunneling in plasmonic gaps: extending the quantum corrected model to practical situations. Faraday discussions, 178, 151-183
